# High-Throughput Screening System Evaluation of *Andrographis paniculata* (Burm.f.) Extracts and Their Fractions against Mosquito Vectors

**DOI:** 10.3390/insects15090712

**Published:** 2024-09-18

**Authors:** Patcharawan Sirisopa, Theerachart Leepasert, Thitinun Karpkird, Jirod Nararak, Kanutcharee Thanispong, Alex Ahebwa, Theeraphap Chareonviriyaphap

**Affiliations:** 1 Department of Entomology, Faculty of Agriculture, Kasetsart University, Bangkok 10900, Thailand; golf.patcharawan@gmail.com (P.S.); fagrjrn@ku.ac.th (J.N.); alex.ahebwa14@gmail.com (A.A.); 2 Department of Chemistry, Faculty of Science, Kasetsart University, Bangkok 10900, Thailand; fscitcl@ku.ac.th (T.L.); fscitnm@ku.ac.th (T.K.); 3 Research and Lifelong Learning Center for Urban and Environmental Entomology, Kasetsart University Institute for Advanced Studies, Kasetsart University, Bangkok 10900, Thailand; 4 Division of Vector Borne Diseases, Department of Disease Control, Ministry of Public Health, Nonthaburi 11000, Thailand; kanut47@gmail.com

**Keywords:** mosquito repellent, *Aedes aegypti*, *Anopheles minimus*, *Anopheles dirus*, spatial repellency, contact irritancy, toxicity, fractionation

## Abstract

**Simple Summary:**

The growing importance of *Andrographis paniculata* as a mosquito repellent has attracted the attention of many researchers. However, its implementation requires robust experimentations to ascertain its efficacy. In this study, the high-throughput screening system was used to evaluate the repellent efficacy of *Andrographis paniculata* crude extract and its fractions against *Aedes aegypti*, *Anopheles minimus*, and *An. dirus*. Both the crude extracts showed repellent activities against all the tested mosquito species. These findings suggest that fractionation of *A. paniculata* extracts is valuable to assessing their spatial repellent efficacy against mosquitoes. Fractions F3 and F5 hold promise as natural mosquito repellents and could contribute to developing effective mosquito control strategies.

**Abstract:**

Infectious diseases that cause illness and/or death in humans can be contracted from mosquito bites. A viable and alternate method of personal protection that can lower the danger of human exposure to mosquito-borne diseases is the use of plant-based repellents. Using a high-throughput screening system, the current work examined the toxicity, contact irritancy, and spatial repellency of *Andrographis paniculata* crude extract and its fractions against *Aedes aegypti*, *Anopheles minimus*, and *An. dirus*. Five fractions (i.e., F1, F2, F3, F4, and F5) were separated from the crude extract by column and thin layer chromatography and analyzed using high-performance liquid chromatography and mass spectrometry. The major active compounds identified from F3 and F5 were 4-deoxy-11,12-didehydroandrographolide and andrographolide. Three concentrations (1.0, 2.5, and 5.0%) for each of the crude extracts and the five fractions were individually impregnated on nylon netting strips and evaluated against the three mosquito species. Results showed that the highest contact irritancy was elicited by the crude extract at 5% concentration against *Ae. aegypti* (43.70% escaped). Results of the spatial activity index (SAI) showed that fractions F3 and F5 at 2.5% demonstrated the strongest repellency against *Ae. aegypti* (SAI = 0.84) and *An. minimus* (SAI = 0.83), respectively. Both the crude extract and its components did not cause any knockdown or mortality. These findings suggest that fractionation of *A. paniculata* extracts is valuable in assessing their spatial repellent efficacy against mosquitoes. Fractions F3 and F5 hold promise as natural mosquito repellents and could contribute to developing effective mosquito control strategies.

## 1. Introduction

Mosquitoe-borne diseases present a great burden to health systems in many regions of the world. Malaria, dengue, chikungunya, Zika and yellow fever are some of the colossal threats to human health, transmitted by some mosquitoes of the genera of *Aedes* and *Anopheles* [1,2]. Hematophagous mosquitoes require a blood meal to accomplish their development needs. During blood-feeding, they can transmit the disease-causing pathogenic viruses and parasites into the human bloodstream [3,4]. In Thailand, where *Aedes aegypti* (L.) the principal vector of dengue is endemic [5], 158,000 dengue cases were recorded in 2023 [6]. On the other hand, *Anopheles minimus* Theobald and *An dirus* Peyton & Harrison which predominantly inhabit the forested and mountainous areas of the country, have maintained a slow but continuous transmission of cross-border malaria [7,8,9]. In spite of these challenges, after adopting the National Malaria Elimination Strategy 2017–2026 in 2016 and instituting the 1-3-7 surveillance approach, malaria has declined significantly [10]. Robust mosquito control strategies and research have crucially made this progress possible. Mosquito control consists of adulticiding, larviciding, environment management, personal protection with topical repellents and biological control [6,11]. The major challenge in using chemical control has been insecticide resistance in most disease vectors [11]

The application of topical mosquito repellents is a useful strategy for reducing mosquito bites and, consequently, the spread of diseases by offering individual protection. The most powerful active ingredient in topical repellents is DEET (N, N-diethyl-3-methylbenzamide), which works extremely well against a variety of biting insects. Finding more organically derived and environmentally friendly repellents is necessary, though, as DEET intoxication has been linked to skin irritation, allergic reactions, and, in the worst cases, neurological abnormalities [12,13]. Additionally, decreased sensitivity has been observed in *Ae. aegypti* mosquitoes that were previously exposed to DEET [14]. This attempt to find synthetic alternatives matches the increasing demand from consumers for eco-friendly products. Research has shown that several under utilized plant extracts and essential oils are efficient against a wide range of mosquito species [15,16,17,18]. However, to rival DEET, researchers must conduct a buttress of experiments to reveal the effectiveness and safety of botanical products. The effectiveness of topical repellents can be evaluated in a laboratory setting using a variety of techniques. These include the arm-in-cage test, the Y-tube olfactometer, the excito-repellency system (ER), and the high throughput screening system (HITSS) [19,20]. Some of these have been used to evaluate the effectiveness of novel products made with traditional DEET, especially in the case of plant-based alternatives. Using HITSS, researchers assessed how *An. minimus* and *Ae. aegypti* responded to DEET and several essential oils. The results, which were reported as spatial activity indices, revealed that citronella oil and DEET had comparable repellent properties [21]. Some researchers have used the ERS to compare the repellent efficacy of DEET with that of EOs from the roots of vetiver grass (*Vetiver zizanioides* (L.)), the flowers of ylang ylang (*Cananga odorata* Hook. F. & Tomson), and ethanolic crude extracts of *Andrographis paniculata* (Burm.f.) Wall. ex Nees leaves. Their results demonstrated closely comparable contact irritancy and non-contact spatial repellency effects against the laboratory and field strains of *Culex quinquefasciatus* Say [22]. These studies have shown that there are potential alternatives to DEET that are widely available and acceptable. In addition to simple extraction methods such as hydro-distillation [23], plant-based repellents are generally regarded as safe [15].

*Andrographis paniculata* (Lamiales: Acanthaceae), commonly known as the ‘King of Bitters’ [24], exhibits a broad spectrum of pharmacological properties. Research suggests that it may have anticancer, anti-HIV, antihyperglycemic, and antimalarial effects [25,26,27,28]. Notably, the plant’s characteristic bitter taste, attributed mainly to the compounds andrographolide and kalmeghin, may also play a role in its apparent ability to deter insects [29,30]. Numerous investigations have shown that *A. paniculata* extracts exhibit insecticidal and repellent activities against agricultural pests and medically important insects. When applied on human skin, the extracts have demonstrated repellent effects against *Cx tritaeniorhynchus* Giles and *An stephensi Liston* [31,32,33]. When tested in a WHO tube test, the chloroform extract of *A. paniculata* leaves was found to have a strong adulticidal efficacy against *An. subpictus* Grassi [34] and *An. stephensi* [35]. Elango et al. [36] demonstrated that hexane and methanolic extracts from *A. paniculata* leaves could eradicate fourth-instar larvae of *An. subpictus* and *Cx. tritaeniorhynchus*. In another study, ethanolic and methanolic extracts delayed larval development and reduced the longevity and fecundity of female *An. stephensi* [37]. In vitro studies have also indicated that flavones, a flavanone and a diterpenoid (14-deoxy-11,12-didehydroandrographolide) from *A. paniculata* can inhibit CYP6AA3 and CYP6P7 insecticide detoxifying enzymes of *An. minimus* [38]. Although *A. paniculata* shows potential as an all-round control for vector-borne diseases, there is not enough data to validate its public usage. Its repellent potential and that of its components are not yet well studied.

The current study was designed to evaluate the repellent and insecticidal activity of *A. paniculata* extract and its fractions to be used as a natural repellent against mosquitoes. Utilizing the high-throughput screening system (HITTS), we examined three crucial aspects of mosquito behavior in response to *A. paniculata*: contact irritancy, repellency, and toxicity. This research specifically considered three mosquito species with principal public health implications within Thailand: *Ae. aegypti*, *An. dirus*, and *An. minimus*.

## 2. Materials and Methods

### 2.1. Andrographis paniculata Crude Extract

The crude extract of *A. paniculata* leaves was purchased from Thai China Flavours and Fragrances Industry Co., Ltd., Ayutthaya, Thailand (Batch No. 19121204-1). The ethanolic extract was then fractionated using the column chromatography technique and analyzed by HPLC-MS [39].

Fractionation was accomplished by dissolving 65 g of the crude extract in ethyl acetate and mixing it with silica gel (100–200 mesh). Ethyl acetate was removed from the mixture by using a rotary evaporator, loaded onto the column, and gradient-eluted using 80% ethyl acetate in hexane to 100% ethyl acetate. The fractions were collected and combined, monitored with thin-layer chromatography, and consolidated into five fractions: F1, F2, F3, F4, and F5. Three concentrations, i.e., 1.0, 2.5, and 5.0% (*w*/*v*) were evaluated for every fraction and the crude extract, against the three mosquito species.

### 2.2. Mosquitoes

This study was conducted with three insecticide-susceptible laboratory mosquito species: *An. minimus*, *An. dirus* and *Ae. aegypti*. Both *An. minimus* (KU strain) and *An. dirus* (TMMU strain) were originally collected from the field in Thailand [40], whereas *Ae. aegypti* (USDA strain) was provided by the US Department of Agriculture. All the species were maintained in an insectary at the Department of Entomology, Faculty of Agriculture, Kasetsart University, Bangkok.

### 2.3. Mosquito Rearing

Larvae were maintained in 2–3 L of de-chlorinated filtered tap water inside 4-litre trays. The larvae of *An. minimus* and *An. dirus* received a finely ground powder of fish food flakes (TetraMin, TetraGmbH, Melle, Germany) three times a day until pupation [41]. However, *Ae. aegypti* larvae received standard fish food pellets (Floating Fish Food Pellets, PondMax, Forrestdale, WA 6112, Australia) in equal amounts of two pellets, approximately 100–150 mg, per tray per day, until pupation [42]. Water surface scum arising from food remains was removed daily using tissue paper. The water was changed after every two to three days. Pupae were collected daily into white plastic bowls (250 mL) and placed in 30 × 30 × 30 cm wire mesh cages with metallic frames for adult eclosion. Adult mosquitoes were given 10% (*w*/*v*) sucrose in water ad libitum. Human blood was provided using an artificial membrane feeding system, to support mosquito egg production [43]. The system uses glass feeders connected to a circulating water bath by rubber tubing. The water bath was maintained at 37 °C throughout each feeding cycle. Fresh pig intestines were used as feeding membranes. The membranes were fasted by rubber bands across the bottom of the glass feeders. Blood was added to the glass feeders to pool at the bottom and was then carefully placed above mosquito-rearing cages to facilitate feeding [43]. Pathogen-free human blood was obtained from the Thai Red Cross Society and handled in the KU insectary following a written standard operating procedure. The maintenance of the *An. dirus* colony required the application of artificial insemination technique to induce mating [41]. Inside the insectary, ambient conditions were maintained at 25 ± 2 °C, 80 ± 10% relative humidity and a 12 L:12 D photoperiod.

### 2.4. Netting Strip Impregnation

*Andrographis paniculata*’s crude extract and its fractions were first diluted with absolute ethanol to make a 5% (*v*/*w*) treatment stock solution and were then serially diluted to final three concentrations 1%, 2.5%, and 5%. Nylon netting was cut into equal-sized strips measuring 25 cm × 11 cm, length × width. A hand-held 2 mL glass pipette was used to evenly apply 1.5 mL of the treatment solution onto each strip [44]. Ethanol alone was used to coat the control strips. All strips were dried at room temperature for at least 1 h before the assay. Each strip was used only once and discarded after.

### 2.5. High-Throughput Screening System (HITSS)

HITSS is approved by the WHO as a yardstick for evaluating repellents [19]. Comprising three tests—toxicity, contact irritancy, and spatial repellency—the HITSS provides a comprehensive evaluation of the efficacy of repellent products. The full operationalization of the system was described in detail by Grieco et al. [20]. The device consists of three main parts, i.e., the aluminum control and treatment cylinders and the clear Plexiglas cylinder [20]. The treatment cylinder consists of a treatment drum with a treated material (fabric or filter paper) attached to it acting as the exposure chamber. The control cylinder consists of a treatment drum with an untreated material (fabric or filter paper) attached to it acting as the control chamber. Depending on the assay, either some or all three parts may be interconnected. In the present study, test mosquitoes were 3–5 day-old females that had not taken a blood meal before and had been sugar-starved for 24 h prior to the test.

#### 2.5.1. Contact Irritancy Test

Following physical contact, the sense of touch induces a shift away from the treated surface, a response known as contact irritancy [45]. In the contact irritancy test, the exposure/control chamber was connected to the clear cylinder using a linking section. The linking section consists of a butterfly valve that can be set in a closed or opened position [20]. After assembling the parts, 10 mosquitoes were transferred into the exposure chamber. After 30 s of acclimatization, the butterfly valve was opened and closed again after 10 min. The number of mosquitoes remaining in the exposure chamber (non-escapes) and those in the clear cylinder (escapes) were recorded. The treatment and control assays were conducted simultaneously. Between each replication, the mosquitoes were aspirated from the exposure/control chambers and the clear cylinders. A total of six replications (utilizing 60 female mosquitoes in total) were conducted for each treatment (*n* = 6) and each concentration (n = 3).

#### 2.5.2. Spatial Repellency Assay

In the spatial repellency test, the mosquito is elicited to move away from the treated surface without making physical contact [45]. To assemble a spatial repellency assay system, the treatment and control chambers were connected to opposite sides of the clear cylinder using the linking section [20]. With the butterfly valves in both linking sections closed, 20 mosquitoes were transferred into the clear cylinder. After 30 s of acclimatization, the butterfly valves were simultaneously opened and again closed after 10 min. The number of mosquitoes in the treatment and control chambers as well as the clear cylinder were recorded. A total of nine replications (utilizing 180 female mosquitoes in total) were conducted for each treatment (n = 6) and each concentration (n = 3).

#### 2.5.3. Toxicity

The toxicity test evaluates the knockdown and/or death of the mosquitoes after making physical tarsal contact with the chemical-treated surface. Twenty female mosquitoes were aspirated into the exposure chamber containing the nylon strip treated with either the crude extract or its fractions—F1, F2, F3, F4, and F4. Simultaneously, twenty female mosquitoes were transferred into the control chamber containing the nylon strip treated with absolute ethanol. After one hour, the number of knocked down mosquitoes was recorded. Then all mosquitoes were transferred into holding paper cups and provided with a 10% sugar solution with soaked cotton balls. The cups were transferred to the insectary and checked again after 24 h for mortality. The same number of replications as in the contact irritancy test was conducted.

### 2.6. Data Analysis

All data were initially input and cleaned in Microsoft office excel and exported into the SAS Institute program [46] for analysis. Contact irritancy data were analyzed using the two-sample Wilcoxon test to assess the variance in the number of mosquitoes escaping from treated versus control cylinders. For spatial repellency data, the Wilcoxon signed-ranks test was used to compare the mean Spatial Activity Index (SAI) for each chemical.

$$SAI=\frac{\left[Nc\;–Nt\;\right]}{\left[Nc+Nt\right]}\times \frac{[Nc+Nt]\;}{\left[N\right]}\;$$

where *Nc* is the number of mosquitoes in the control chamber and *Nt* is the number in the treated chamber, *N* is the total number of mosquitoes in the test [19]. The SAI values range from −1 to 1, where 0 indicates no response, −1 signifies attraction (mosquitoes move to the treatment chamber), and 1 means repellency (more mosquitoes move to the control chamber) [19].

### 2.7. HPLC-MS Analysis

The two fractions F3 and F5 of the five consolidated from the crude extract of *A. paniculata*, having demonstrated a superior repellency efficacy, were further analyzed using high-performance liquid chromatography and mass spectrometry (HPLC-MS). Using HPLC-grade methanol and acetonitrile (RCI Labscan, Bangkok, Thailand) in negative-ion mode acquired from an electrospray ionization source of the HPLC-MS system (6490 Triple Quadrupole, Agilent Technologies, CA, USA) was critical to the process. Separation was performed on a ZORBAX Eclipse plus C18 rapid resolution column (4.6 mm × 100 mm, 3.5 micron) (Agilent Technologies). The mobile phase was a mixture of two eluents, water and acetonitrile (ACN), delivered at a flow rate of 0.5 mL/min using a gradient system (0–6 min, 35% ACN; 6–12 min, 35–45% ACN; 12–13 min, 45–35% ACN). The crude extract had a concentration of 125 ppm (0.0125%) in methanol. The reference standard was Andrographolide (purity 98%, Sigma Aldrich, St. Louis, MO 63118, USA) [39].

## 3. Results

### 3.1. Isolating and Fractionating Crude Extract

In this study, an ethanolic crude extract of *A. paniculata* was separated by flash column chromatography, resulting in five fractions. This process separates the plant’s various compounds based on their chemical polarities and facilitates the identification of an extract with specific biological activity. The largest yield was found in the F5 fraction, 3.93 g. The yields of fractions F1, F2, F3, and F4 were 1.21 g, 0.90 g, 2.95 g and 0.56 g, respectively.

### 3.2. High Throughput Screening System

**Figure 1:**
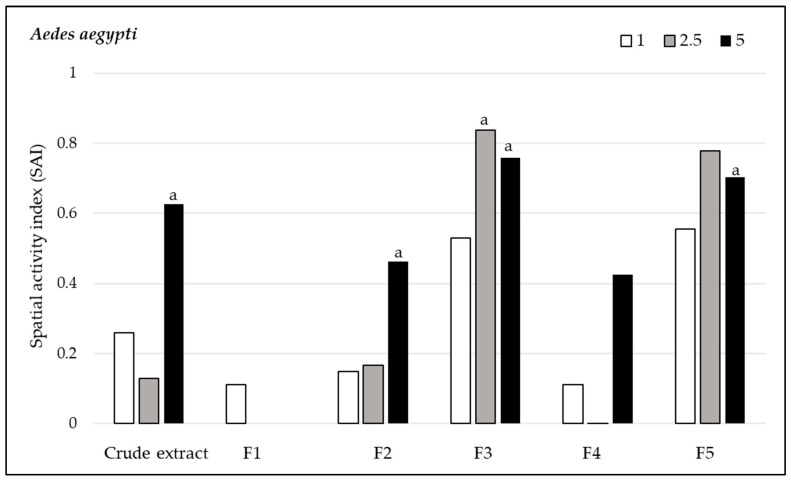
Spatial repellent activity of *Andrographis paniculata* crude extract and its fractions at 1%, 2.5% and 5% concentrations tested against *Aedes aegypti*. a, denotes statistically significant (signed rank test, *p* < 0.05) repellent response compared with matched controls.

**Figure 2:**
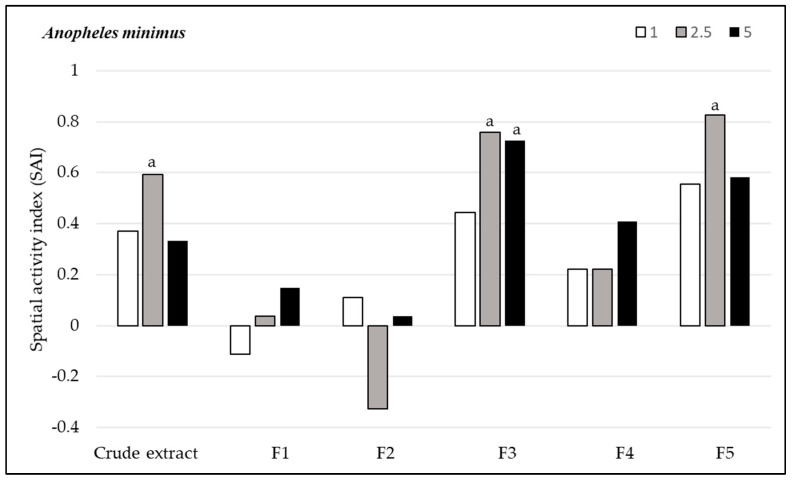
Spatial repellent activity of *Andrographis paniculata* crude extract and its fractions at 1%, 2.5% and 5% concentrations tested against *Anopheles minimus*. a, denotes statistically significant (signed rank test, *p* < 0.05) repellent response compared with matched controls.

**Figure 3:**
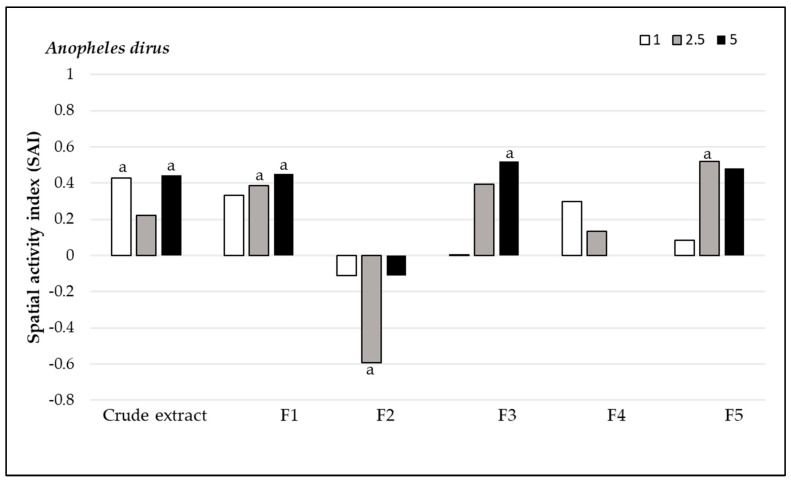
Spatial repellent activity of *Andrographis paniculata* crude extract and its fractions at 1%, 2.5% and 5% concentrations tested against *Anopheles dirus*. a, denotes statistically significant (signed rank test, *p* < 0.05) repellent response compared with matched controls.

The irritancy, repellency, and toxicity effects of *A. paniculata’s* crude extract and fractions were evaluated using the HITSS method against *Ae. aegypti*, *An minimus*, and *An.dirus*. Responses of the unfed female mosquitoes are detailed in Table 1, Table 2 and Table 3 and Figure 1, Figure 2 and Figure 3. A supplementary file has been provided for toxicity data (Table S1).

#### 3.2.1. Contact Irritancy

The investigation on the degree of behavioral escape response to extracts of *A. paniculata* revealed significant variations among different species of mosquitoes and concentration levels of extracts. *Aedes aegypti* consistently displayed a higher contact irritancy reaction compared to *An. minimus* and *An. dirus*. The most notable response in *Ae. aegypti* was observed at a concentration of 5% crude extract resulting in a 43.70 percent escape. Within the fractions, the greatest percentages of contact irritancy responses were identified in fractions F3 and F5 at a concentration of 5%, exhibiting moderate irritancy responses of 30.9 and 21.67 percent escape (*p* < 0.05), respectively. For *An. minimus* and *An. dirus*, minimal irritancy responses were observed across all concentrations of the crude extract and fractions, with less than 20% escape response documented (Table 2 and Table 3).

**Table 1:** Escape response of *Aedes aegypti* in the contact irritancy assay to crude extract and fraction of *Andrographis paniculata*.

Repellent	Concentration (%, *w*/*v*)	R (N)	Number Escaping (Mean ± SE per Replicate)		Percent Escaping (Mean ± SE)	*p*-Value ^b^
			Treated	Control ^a^		
Crude extract	1	6 (60)	3.00 ± 0.26	0.50 ± 0.22	26.11 ± 3.11	**0.0022**
	2.5	6 (60)	4.00 ± 0.86		36.48 ± 9.75	**0.0087**
	5	6 (60)	4.67± 0.33		43.70 ± 3.87	**0.0022**
F1	1	6 (60)	0.83 ± 0.31	0.17 ± 0.17	6.48 ± 4.36	0.1970
	2.5	6 (60)	1.17 ± 0.17		10.00 ± 2.58	**0.0130**
	5	6 (60)	1.00 ± 0.26		8.33 ± 3.07	0.0671
F2	1	6 (60)	0.83 ± 0.17	0.33 ± 0.21	5.00 ± 2.23	0.2424
	2.5	6 (60)	1.33 ± 0.21		10.00 ± 3.65	**0.0325**
	5	6 (60)	1.17 ± 0.31		8.15 ± 4.92	0.1234
F3	1	6 (60)	1.50 ± 0.22	0.33 ± 0.21	11.85 ± 3.06	**0.0216**
	2.5	6 (60)	3.00 ± 0.52		27.78 ± 4.62	**0.0065**
	5	6 (60)	3.33 ± 0.21		30.9 ± 2.35	**0.0022**
F4	1	6 (59)	0.67 ± 0.21	0.67 ± 0.21	1.67 ± 3.32	1.0000
	2.5	6 (60)	1.33 ± 0.33		7.04 ± 3.41	0.2381
	5	6 (60)	1.50 ± 0.22		8.70 ± 3.12	**0.0758**
F5	1	6 (60)	1.00 ± 0.25	0.00 ± 0.00	8.33 ± 3.07	**0.0152**
	2.5	6 (60)	1.83 ± 0.60		17.22 ± 5.26	**0.0152**
	5	6 (60)	2.17 ± 0.17		21.67 ± 1.67	**0.0022**

^a^ One control was maintained for the three concentrations for each fraction and the crude extract. ^b^ *p*-value < 0.05 (in bold font) indicates a significant difference between the number escaping in the treatment chamber and the control chamber. R = number of replicates, N = total number of mosquitoes, SE = standard error, *w*/*v* = weight per volume, F1–F5 = fractions of the extract.

**Table 2:** Escape response of *Anopheles minimus* in the contact irritancy assay to crude extract and fraction of *Andrographis paniculata*.

Repellent	Concentration (%, *w*/*v*)	R (N)	Number Escaping (Mean ± SE per Replicate)		Percent Escaping (Mean ± SE)	*p*-Value ^b^
			Treated	Control ^a^		
Crude extract	1	6 (60)	1.17 ± 1.67	0.17 ± 0.17	10.19 ± 0.19	**0.0130**
	2.5	6 (60)	1.67 ± 0.33		15.37 ± 2.42	**0.0087**
	5	6 (60)	1.33 ± 0.21		11.85 ± 1.63	**0.0108**
F1	1	6 (60)	0.17 ± 0.17	0.00 ± 0.00	1.67 ± 1.67	1.0000
	2.5	6 (59)	0.50 ± 0.22		6.67 ± 3.33	0.1818
	5	6 (60)	0.67 ± 0.33		6.67 ± 3.33	0.1818
F2	1	6 (60)	0.33 ± 0.21	0.17 ± 0.17	1.48 ± 3.21	1.0000
	2.5	6 (60)	0.67 ± 0.21		5.00 ± 2.24	0.2424
	5	6 (60)	0.67 ± 0.21		5.00 ± 2.24	0.2424
F3	1	6 (60)	1.00 ± 0.26	0.33 ± 0.21	6.48 ± 4.36	0.1775
	2.5	6 (59)	1.33 ± 0.21		13.33 ± 4.21	**0.0325**
	5	6 (60)	1.50 ± 0.22		11.67 ± 4.01	**0.0216**
F4	1	6 (59)	0.17 ± 0.17	0.17 ± 0.17	1.48 ± 3.21	1.0000
	2.5	6 (60)	0.50 ± 0.22		3.15 ± 3.48	0.5455
	5	6 (60)	0.50 ± 0.22		3.15 ± 3.48	0.5455
F5	1	6 (60)	0.83 ± 0.31	0.33 ± 0.21	5.00 ± 3.42	0.4048
	2.5	6 (59)	1.50 ± 0.34		13.52 ± 5.56	**0.0325**
	5	6 (60)	1.67 ± 0.33		13.70 ± 3.50	**0.0216**

^a^ One control was maintained for the three concentrations for each fraction and the crude extract. ^b^ *p*-value < 0.05 (in bold font) indicates a significant difference between the number escaping in the treatment chamber and the control chamber. R = number of replicates, N = total number of mosquitoes, SE = standard error, *w*/*v* = weight per volume, F1–F5 = fractions of the extract.

**Table 3:** Escape response of *Anopheles dirus* in the contact irritancy test to crude extract and fraction of *Andrographis paniculata*.

Repellent	Concentration (%, *w*/*v*)	R (N)	Number Escaping (Mean ± SE per Replicate)		Percent Escaping (Mean ± SE)	*p*-Value ^b^
			Treated	Control ^a^		
Crude extract	1	6 (60)	1.00 ± 0.26	0.17 ± 0.17	8.52 ± 1.71	0.0671
	2.5	6 (60)	1.33 ± 0.22		11.67 ± 3.07	**0.0108**
	5	6 (60)	1.67 ± 0.33		15.00 ± 4.28	**0.0087**
F1	1	6 (60)	0.50 ± 0.22	0.17 ± 0.17	3.33 ± 2.11	0.5455
	2.5	6 (60)	0.50 ± 0.22		3.33 ± 2.11	0.5455
	5	6 (60)	0.83 ± 0.31		6.67 ± 3.33	0.1970
F2	1	6 (60)	0.50 ± 0.34	0.17 ± 0.17	3.15 ± 4.33	0.7273
	2.5	6 (60)	0.67 ± 0.21		5.00 ± 2.24	0.2424
	5	6 (60)	0.83 ± 0.17		6.67 ± 2.11	**0.0801**
F3	1	6 (60)	0.83 ± 0.31	0.50 ± 0.22	3.52 ± 2.23	0.6753
	2.5	6 (60)	1.17 ± 0.31		6.67 ± 4.40	0.2316
	5	6 (60)	1.50 ± 0.22		10.37 ± 2.59	**0.0433**
F4	1	6 (60)	0.17 ± 0.17	0.17 ± 0.17	−0.19 ± 2.73	1.0000
	2.5	6 (60)	0.17 ± 0.17		−0.19 ± 2.73	1.0000
	5	6 (60)	0.67 ± 0.21		5.00 ± 2.24	0.2424
F5	1	6 (60)	0.67 ± 0.21	0.33 ± 0.21	3.33 ± 2.10	0.5671
	2.5	6 (59)	1.33 ± 0.33		12.04 ± 1.61	0.0801
	5	6 (60)	1.50 ± 0.34		11.67 ± 4.77	**0.0325**

^a^ One control was maintained for the three concentrations for each fraction and the crude extract. ^b^ *p*-value < 0.05 (in bold font) indicates a significant difference between the number escaping in the treatment chamber and the control chamber. R = number of replicates, N = total number of mosquitoes, SE = standard error, *w*/*v* = weight per volume, F1F5 = fractions of the extract.

#### 3.2.2. Spatial Repellency

*Andrographis paniculata* extracts demonstrated varying levels of spatial repellency against the three mosquito species. Against *Ae. aegypti* populations, all compounds, except for fraction F1 at 2.5% concentration (SAI = 0) and fraction F4 at 2.5% concentration (SAI = −0.01), showed positive spatial activity index values, with no statistically significant variances (*p* > 0.05). The highest spatial repellency against *Ae. aegypti* was observed in fraction F3 at a concentration of 2.5% (SAI = 0.84), followed by fraction F5 with the same concentration (SAI = 0.78), both showing significant differences from the control group (*p* < 0.05). The crude extract showed significant spatial repellency (SAI = 0.63, *p* < 0.05) at 5.0% concentration (Figure 1). Regarding *An. minimus*, all compounds, except for fraction F1 at 1% concentration (SAI = −0.11) and F2 at 2.5% concentration (SAI = −0.32), displayed positive spatial activity index values, with no significant differences (*p* > 0.05) from control groups. The highest repellent response was produced at a 2.5% concentration by F5 (SAI = 0.83), followed by F3 (SAI = 0.75), and the crude extract showed SAI = 0.59, all of which were significantly different compared to the control (*p* < 0.05) (Figure 2). *Anopheles dirus* exhibited a positive repellent response to all compounds except F2. The highest spatial repellent response was exhibited by F3 at 5% (SAI = 0.52) and F5 at 2.5% concentration (SAI = 0.52), with statistically significant differences when compared to the control group (*p* < 0.05) (Figure 3). In summary, F3 and F5 demonstrated the most promising spatial repellent index against the tested mosquitoes.

### 3.3. Toxicity Assay

Toxicity efficacy data of the crude extract and various fractions derived from *A. paniculata* at concentrations of 1%, 2.5%, and 5% were evaluated against *Ae. aegypti*, *An. minimus*, and *An. dirus* in the current investigation. Nevertheless, no knockdown and very minimal mortality (at most, one mosquito per treatment and/or concentration) was observed across all experiments (Table S1). The findings indicate that the crude extract and fractions derived from *A. paniculata* did not exhibit substantial toxicity toward the tested mosquitoes.

### 3.4. HPLC-MS Analysis

**Figure 4:**
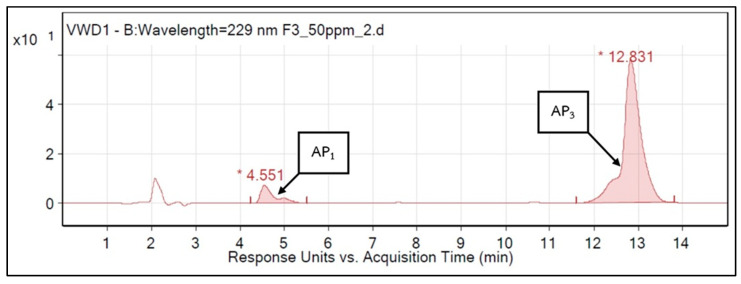
HPLC-MS chromatogram of fraction 3 (AP1 = Andrographolide 8.52%, AP3 = 14-Deoxy-11,12didehydroandrographolide 91.48%). * Acquisition times for AP1 and AP3.

**Figure 5:**
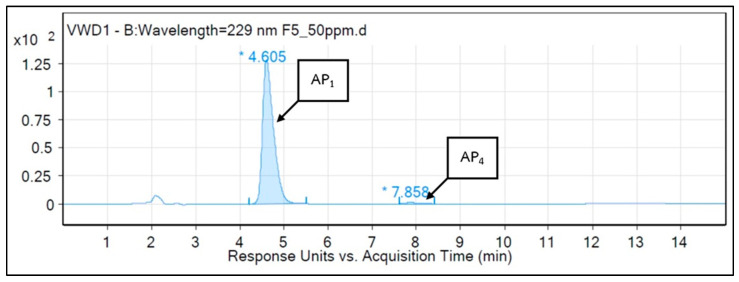
HPLC-MS chromatogram of fraction 5 (AP1 = Andrographolide 98.97%, AP4 = Neoandrographolide 1.03%). * Acquisition times for AP1 and AP3.

According to above results on repellency and irritancy, fractions F3 and F5 showed the highest repellent activities against *Ae. aegypti*, *An. minimus*, and *An. dirus*. The main active compounds of *A. paniculata* in F3 and F5 were identified by using HPLC-MS. Analysis of F3 (see Figure 4) showed andrographolide, AP1 (8.52%) and 14-Deoxy-11,12-didehydroandrographolide, AP3 (91.48%) as the main compounds with acquisition times of 4.55 and 12.83 min, respectively. In F5 (Figure 5), the predominant compounds were andrographolide, AP1 (98.97%) and neoandrographolide, AP4 (1.03%), with acquisition times of 4.605 and 7.858 min, respectively.

## 4. Discussion

For centuries, traditional practices have been using medicinal plants and their products to drive away nuisance insects [15]. Repellents are the most common intervention methods for guarding against host-seeking mosquitoes in the community [47]. Some plant extracts, such as essential oils or terpenes, have been demonstrated to possess a natural repellent effect against various mosquito species and arthropod pests [48,49,50]. Although their effectiveness has been debated and further research recommended, their low toxicity and environmental benefits are the reasons why they can be a good choice to consider when developing repellents for mosquitoes [15,50,51].

*Andrographis paniculata*, commonly known as the “king of bitters”, has recently emerged as a promising natural mosquito repellent [30]. This medicinal plant, native to India and Southeast Asia, has a long history of traditional use in mosquito control. It harbors a diverse array of bioactive compounds, including andrographolide (AP1), neoandrographolide (AP4), 14-Deoxy-11,12-didehydroandrographolide (AP3), and various diterpenoids [30]. These compounds have been documented to exhibit insecticidal, larvicidal, and repellent properties against mosquito vectors [52,53,54]. Numerous studies have demonstrated the efficacy of *A. paniculata* extracts in repelling adult mosquitoes, including *Ae. aegypti*, *Culex quinquefasciatus*, *Cx. tritaeniorhynchus*, *An. stephensi*, and *An. subpictus* when applied to human skin [31,32,33,52,55]. When mixed with essential oils from the roots of vetiver grass (*Vetiver zizanoides*) and the flowers of ylang-ylang (*Cananga odorata*), the mixtures were reported to repel *Cx. quinquefasciatus* better than the gold standard repellent DEET in an excito-repellency system [22]. In a recent study, hexane, chloroform, and methanol crude extracts of *A. paniculata* leaves were evaluated against *Ae. aegypti* and *Cx. quinquefasciatus* mosquitoes. The methanolic extract was then fractionated by using thin-layer chromatography. Out of the 10 consolidated fractions, fraction 4 exhibited remarkable larvicidal and pupicidal activities against the larvae, pupae, and eggs [55].

The current study investigated the contact irritancy, spatial repellency, and toxicity of *A. paniculata* crude extract and its fractions (F1, F2, F3, F4, and F5) against three mosquito species (*Ae. aegypti*, *An. minimus*, and *An. dirus*) by using a high-throughput screening system (HITSS). The results revealed that *Ae. aegypti* exhibited the highest sensitivity showing moderate contact irritancy against the crude extract and substantial spatial repellency against fractions F3 and F5. This finding aligns with previous research, where *A. paniculata* extracts demonstrated repellent activity against *Ae. aegypti* [55,56]. Sukkanon et al. [57] evaluated the efficacy of *A. paniculata* for its noncontact repellency, contact ex-citation (irritancy + repellency), and knockdown/toxicity response against five species of mosquitoes by using an excito-repellency assay system under laboratory-controlled conditions. Their findings indicated that *A. paniculata* had relatively higher spatial repellency action against day-biting *Ae. aegypti* and *Ae. albopictus* than night-biting *An. dirus*, *An. epiroticus*, and *Cx. quinquefasciatus*.

Although *A. paniculata* extracts showed promise against *Ae. aegypti*, their efficacy against *Anopheles* spp. was more limited. This variation in repellency could be attributed to differences in olfactory receptor sensitivities and preferences for specific compounds present in the extract among these mosquito species. For example, essential oil molecules can bind to the mosquito’s olfactory receptors, effectively disrupting the insect’s ability to detect host-seeking chemical signals and reducing bites. Alternatively, the interaction of essential oil molecules with these receptors could trigger a repellent response in the mosquito, causing it to avoid the treated area or individual. The precise mechanism by which essential oils disrupt the mosquito’s olfactory system may vary depending on the specific compounds present in the oil and their interactions with the complex array of receptors involved in host-seeking behavior [57,58]. Furthermore, the timing of the experiments, conducted between 09:00 and 16:30 h, may have influenced the results, as different mosquito species exhibit varying activity levels throughout the day due to their circadian rhythms [59]. To fully understand the repellent properties of *A. paniculata*, further research is necessary to identify the specific compounds responsible for repellency and optimize their formulation to enhance efficacy against a wider range of mosquito species.

The identification of 14-deoxy-11,12-didehydroandrographolide (AP3) and andrographolide (AP1) as the primary active compounds in fractions F3 and F5, respectively, through HPLC-MS analysis is consistent with previous studies highlighting the presence of these compounds in *A. paniculata* [60]. These compounds are known for their diverse biological activities, including insecticidal properties [61]. In this study, the lack of knockdown or mortality in the toxicity assay suggests that the crude extract and fractions of *A. paniculata* may not possess acute toxic effects against the tested mosquito species. This result is consistent with the findings of Sukkanon et al. [57] who also reported no knockdown or mortality 24 h post-exposure using an excito-repellency assay system. However, further research is warranted to evaluate these extracts’ potential sublethal effects or delayed toxicity.

## 5. Conclusions

This study demonstrates the potential of *A. paniculata*, particularly fractions 3 and 5, as a source of natural mosquito repellents, especially against *Ae. aegypti* and *An. minimus*. Identifying the main active compounds contributes to our understanding of the plant’s chemical composition and potential for developing effective and environmentally friendly mosquito control strategies. Further research should focus on evaluating these compounds’ long-term efficacy and safety, as well as exploring their mechanisms of action and potential synergistic effects with other natural repellents.

## Data Availability

All data have been included in the submitted manuscript.

## Supplementary Materials

The following supporting information can be downloaded at: https://www.mdpi.com/article/10.3390/insects15090712/s1, Table S1: Toxicity of test compounds at different concentrations.

## References

[1] World Health Organization (2014). A Global Brief on Vector-Borne Diseases.

[2] World Health Organization (2023). Global Report on Neglected Tropical Diseases.

[3] Carrington L.B., Simmons C.P. (2014). Human to mosquito transmission of dengue viruses. Front. Immunol..

[4] Churcher T.S., Sinden R.E., Edwards N.J., Poulton I.D., Rampling T.W., Brock P.M., Griffin J.T., Upton L.M., Zakutansky S.E., Sala K.A. (2017). Probability of transmission of malaria from mosquito to human is regulated by mosquito parasite density in naïve and vaccinated Hosts. PLoS Pathog..

[5] Ahebwa A., Hii J., Neoh K.-B., Chareonviriyaphap T. (2023). *Aedes aegypti* and *Aedes albopictus* (Diptera: Culicidae) ecology, biology, behaviour, and implications on arbovirus transmission in Thailand: Review. One Health..

[6] DVBD. Division of Vector-Borne Diseases (2024). Thailand [Internet]. https://lookerstudio.google.com/u/0/reporting/dfa7d4e2-b7f5-48ed-b40a-54f1cd4cbdfb/page/cFWgC.

[7] Tainchum K., Ritthison W., Chuaycharoensuk T., Bangs M.J., Manguin S., Chareonviriyaphap T. (2014). Diversity of *Anopheles* species and trophic behavior of putative malaria vectors in two malaria endemic areas of northwestern Thailand. J. Vector Ecol..

[8] Tananchai C., Pattanakul M., Nararak J., Sinou V., Manguin S., Chareonviriyaphap T. (2019). Diversity and biting patterns of *Anopheles* species in a malaria endemic area, Umphang Valley, Tak Province, western Thailand. Acta Trop..

[9] Zhang C., Yang R., Wu L., Luo C., Yang Y., Deng Y., Wu J., Liu Y., Zhou H. (2022). Survey of malaria vectors on the Cambodia, Thailand, and China-Laos Borders. Malar. J..

[10] Bisanzio D., Sudathip P., Kitchakarn S., Kanjanasuwan J., Gopinath D., Pinyajeerapat N., Sintasath D., Shah J.A. (2024). Malaria Stratification Mapping in Thailand to Support Prevention of Reestablishment. Am. J. Trop. Med. Hyg..

[11] Chareonviriyaphap T., Bangs M.J., Suwonkerd W., Kongmee M., Corbel V., Ngoen-Klan R. (2013). Review of insecticide resistance and behavioral avoidance of vectors of human diseases in Thailand. Parasites Vectors.

[12] Swale D.R., Bloomquist J.R. (2014). Is DEET a dangerous neurotoxicant?. Pest Manag. Sci..

[13] Sudakin D.L., Trevathan W.R. (2003). DEET: A review and update of safety and risk in the general population. J. Toxicol. Clin. Toxicol..

[14] Stanczyk N.M., Brookfield J.F., Field L.M., Logan J.G. (2013). *Aedes aegypti* mosquitoes exhibit decreased repellency by DEET following previous exposure. PLoS ONE.

[15] Maia M.F., Moore S.J. (2011). Plant-based insect repellents: A review of their efficacy, development, and testing. Malar. J..

[16] Rehman J.U., Ali A., Khan I.A. (2014). Plant-based products: Use and development as repellents against mosquitoes: A review. Fitoterapia.

[17] Diaz J.H. (2016). Chemical and plant-based insect repellents: Efficacy, safety, and toxicity. Wilderness Environ. Med..

[18] Moore S.J., Lenglet A., Hill N. (2006). Plant-based insect repellents. Insect Repellents: Principles Methods, and Use.

[19] World Health Organisation (2013). Guidelines for Efficacy Testing of Spatial Repellents.

[20] Grieco J.P., Achee N.L., Sardelis M.R., Chauhan K.R., Roberts D.R. (2005). A novel high-throughput screening system to evaluate the behavioral response of adult mosquitoes to chemicals. J. Am. Mosq. Control Assoc..

[21] Sathantriphop S., Achee N.L., Sanguanpong U., Chareonviriyaphap T. (2015). The effects of plant essential oils on escape response and mortality rate of *Aedes aegypti* and *Anopheles minimus*. J. Vector Ecol..

[22] Boonyuan W., Ahebwa A., Nararak J., Sathantriphop S., Chareonviriyaphap T. (2022). Enhanced excito-repellency of binary mixtures of plant-based mosquito repellents against *Culex quinquefasciatus* say (Diptera: Culicidae), a night biting mosquito species. J. Med. Entomol..

[23] Ahebwa A., Mongkol R., Sawangsri P., Kanjanamaneesathian M. (2020). Vapour-phase efficacy of selected essential oils individually and in combination against *Aspergillus flavus*, *A. niger*, *Fusarium proliferatum*, and *Curvularia lunata*. N. Z. Plant Prot..

[24] Thakur A.K., Chatterjee S.S., Kumar V. (2015). Adaptogenic potential of andrographolide: An active principle of the king of bitters (*Andrographis paniculata*). J. Tradit. Complement. Med..

[25] Kumar S., Singh B., Bajpai V. (2021). *Andrographis paniculata* (Burm.f.) Nees: Traditional uses, phytochemistry, pharmacological properties and quality control/quality assurance. J. Ethnopharmacol..

[26] Xu Y. (2009). Adaptive Immune Response-Modifying and Antimicrobial Properties of *Andrographis paniculata* and Andrographolide. Doctoral Dissertation.

[27] Subramanian R., Zaini Asmawi M., Sadikun A. (2011). A bitter plant with a sweet future? A comprehensive review of an oriental medicinal plant: *Andrographis paniculata*. Phytochem. Rev..

[28] Misra P., Pal N.L., Guru P.Y., Katiyar J.C., Srivastava V., Tandon J.S. (1992). Antimalarial activity of *Andrographis paniculata* (Kalmegh) against *Plasmodium berghei* NK 65 in Mastomys natalensis. Int. J. Pharmacogn.

[29] Akbar S. (2011). *Andrographis paniculata*: A review of pharmacological activities and clinical effects. Altern. Med. Rev..

[30] Hossain M.S., Urbi Z., Sule A., Hafizur Rahman K.M. (2014). *Andrographis paniculata* (Burm.f.) Wall. ex Nees: A review of ethnobotany, phytochemistry, and pharmacology. Sci. World J..

[31] Elango G., Rahuman A.A., Zahir A.A., Kamaraj C., Bagavan A., Rajakumar G., Jayaseelan C., Santhoshkumar T., Marimuthu S. (2010). Evaluation of repellent properties of botanical extracts against *Culex tritaeniorhynchus* Giles (Diptera: Culicidae). Parasitol. Res..

[32] Elango G., Rahuman A.A., Bagavan A., Kamaraj C., Zahir A.A., Rajakumar G., Marimuthu S., Santhoshkumar T. (2010). Efficacy of botanical extracts against Japanese encephalitis vector, *Culex tritaeniorhynchus*. Parasitol. Res..

[33] Govindarajan M., Sivakumar R. (2011). Mosquito adulticidal and repellent activities of botanical extracts against malarial vector, *Anopheles stephensi* Liston (Diptera: Culicidae). Asian Pac. J. Trop. Med..

[34] Elango G., Rahuman A.A., Kamaraj C., Bagavan A., Zahir A.A. (2011). Efficacy of medicinal plant extracts against malarial vector, *Anopheles subpictus* Grassi. Parasitol. Res..

[35] Panneerselvam C., Murugan K. (2013). Adulticidal, repellent, and ovicidal properties of indigenous plant extracts against the malarial vector, *Anopheles stephensi* (Diptera: Culicidae). Parasitol. Res..

[36] Elango G., Rahuman A.A., Bagavan A., Kamaraj C., Zahir A.A., Venkatesan C. (2009). Laboratory study on larvicidal activity of indigenous plant extracts against *Anopheles subpictus* and *Culex tritaeniorhynchus*. Parasitol. Res..

[37] Kuppusamy C., Murugan K. (2010). Effects of *Andrographis paniculata* Nees on growth, development, and reproduction of malarial vector *Anopheles stephensi* Liston (Diptera: Culicidae). Trop. Biomed..

[38] Kotewong R., Duangkaew P., Srisook E., Sarapusit S., Rongnoparut P. (2014). Structure-function relationships of inhibition of mosquito cytochrome P450 enzymes by flavonoids of *Andrographis paniculata*. Parasitol. Res..

[39] Pholphana N., Panomvana D., Rangkadilok N., Suriyo T., Ungtrakul T., Pongpun W., Thaeopattha S., Satayavivad J. (2016). A Simple and Sensitive LC-MS/MS Method for Determination of Four Major Active Diterpenoids from *Andrographis paniculata* in Human Plasma and Its Application to a Pilot Study. Planta Med..

[40] Panthawong A., Sukkanon C., Ngoen-Klan R., Hii J., Chareonviriyaphap T. (2021). Forced egg laying method to establish F1 progeny from field populations and laboratory strains of *Anopheles* mosquitoes (Diptera: Culicidae) in Thailand. J. Med. Entomol..

[41] Sukkanon C., Nararak J., Bangs M.j., Hii J., Chareonviriyaphap T. (2020). Behavioral responses to transfluthrin by *Aedes aegypti*, *Anopheles minimus*, *Anopheles harrisoni*, and *Anopheles dirus* (Diptera: Culicidae). PLoS ONE.

[42] Ahebwa A., Hii J., Neoh K.-B., Leepasert T., Chareonviriyaphap T. (2023). Effects of transfluthrin-treated jute and cotton clothing against resistant and susceptible *Aedes aegypti* (Diptera: Culicidae) in a semifield system. J. Med. Entomol..

[43] Phasomkusolsil S., Tawong J., Monkanna N., Pantuwatana K., Damdangdee N., Khongtak W., Kertmanee Y., Evans B.P., Schuster A.L. (2013). Maintenance of mosquito vectors: Effects of blood source on feeding, survival, fecundity, and egg hatching rates. J. Vector Ecol..

[44] Nararak J., Giorgio C.D., Thanispong K., Sukkanon C., Sanguanpong U., Mahiou-Leddet V., Ollivier E., Chareonviriyaphap T., Manguin S. (2022). Behavioral avoidance and biological safety of vetiver oil and its constituents against *Aedes aegypti* (L.), *Aedes albopictus* (Skuse) and *Culex quinquefasciatus* Say. Curr. Res. Insect Sci..

[45] Achee N.L., Sardelis M.R., Dusfour L., Chauhan K.R., Grieco J.P. (2009). Characterization of spatial repellent, contact irritant, and toxicant chemical actions of standard vector control compounds1. J. Am. Mosq. Control. Assoc..

[46] SAS Institute Inc (1999). SAS Online Doc, Version 8.

[47] Seyoum A., Killeen G., Kabiru E., Knols B., Hassanali A. (2003). Field efficacy of thermally expelled or live potted repellent plants against African malaria vectors in Western Kenya. Trop. Med. Int. Health.

[48] Nerio L.S., Olivero-Verbel J., Stashenko E. (2010). Repellent activity of essential oils: A review. Bioresour. Technol..

[49] George D.R., Finn R.D., Graham K.M., Sparagano O.A. (2014). Present and future potential of plant-derived products to control arthropods of veterinary and medical significance. Parasites Vectors.

[50] Kalita B., Bora S., Sharma A.K. (2013). Plant essential oils as mosquito repellent-a review. Int. J. Res. Dev. Pharm. Life Sci..

[51] Grison C., Carrasco D., Pelissier F., Moderc A. (2020). Reflexion on bio-sourced mosquito repellents: Nature, activity, and preparation. Front. Ecol. Evol..

[52] Prajapati V., Tripathi A.K., Aggarwal K.K., Khanuja S.P. (2005). Insecticidal, repellent and oviposition-deterrent activity of selected essential oils against *Anopheles stephensi*, *Aedes aegypti* and *Culex quinquefasciatus*. Bioresour. Technol..

[53] Vilvest J., Milton M.C.J., Yagoo A. (2023). *Andrographis paniculata* leaf extracts: A natural mosquito control agent in combating *Aedes aegypti* and *Culex quinquefasciatus*. Int. J. Mosq. Res..

[54] Vilvest J., Milton M.C.J., Yagoo A. (2024). Crude extract and effective fractions from *Andrographis paniculata* for immature *Aedes aegypti* and *Culex quinquefasciatus* mosquito. Ecol. Front..

[55] Govindarajan M., Sivakumar R. (2012). Adulticidal and repellent properties of indigenous plant extracts against *Culex quinquefasciatus* and *Aedes aegypti* (Diptera: Culicidae). Parasitol. Res..

[56] Tawatsin A., Wratten S.D., Scott R.R., Thavara U., Techadamrongsin Y. (2001). Repellency of volatile oils from plants against three mosquito vectors. J. Vector Ecol..

[57] Sukkanon C., Karpkird T., Saeung M., Leepasert T., Panthawong A., Suwonkerd W., Bangs M.J., Chareonviriyaphap T. (2020). Excito-repellency activity of *Andrographis paniculata* (Lamiales: Acanthaceae) against colonized mosquitoes. J. Med. Entomol..

[58] Norris E.J., Coats J.R. (2017). Current and future repellent technologies: The potential of spatial repellents and their place in mosquito-borne disease control. Int. J. Environ. Res. Public Health.

[59] Tisgratog R., Sukkanon C., Sugiharto V.A., Bangs M.J., Chareonviriyaphap T. (2021). Time of test periods influence the behavioral responses of *Anopheles minimus* and *Anopheles dirus* (Diptera: Culicidae) to DEET. Insects.

[60] Chao W.W., Lin B.F. (2010). Isolation and identification of bioactive compounds in *Andrographis paniculata* (Chuanxinlian). Chin. Med..

[61] Huang X., Zhang B., Xu H. (2018). Synthesis of andrographolide-related esters as insecticidal and acaricidal agents. Bioorg. Med. Chem. Lett..

